# Drug Repurposing Screening Identifies Novel Compounds That Effectively Inhibit *Toxoplasma gondii* Growth

**DOI:** 10.1128/mSphere.00042-15

**Published:** 2016-03-02

**Authors:** Ashley J. Dittmar, Allison A. Drozda, Ira J. Blader

**Affiliations:** Department of Microbiology and Immunology, SUNY at Buffalo School of Medicine, Buffalo, New York, USA; Indiana University School of Medicine

**Keywords:** drug screens, apicomplexan parasites, host-cell interactions, intracellular pathogens, pharmacology

## Abstract

The urgent need to develop new antimicrobial therapies has spawned the development of repurposing screens in which well-studied drugs and other types of compounds are tested for potential off-label uses. As a proof-of-principle screen to identify compounds effective against *Toxoplasma gondii*, we screened a collection of 1,120 compounds for the ability to significantly reduce *Toxoplasma* replication. A total of 94 compounds blocked parasite replication with 50% inhibitory concentrations of <5 µM. A significant number of these compounds are established inhibitors of dopamine or estrogen signaling. Follow-up experiments with the dopamine receptor inhibitor pimozide revealed that the drug impacted both parasite invasion and replication but did so independently of inhibition of dopamine or other neurotransmitter receptor signaling. Tamoxifen, which is an established inhibitor of the estrogen receptor, also reduced parasite invasion and replication. Even though *Toxoplasma* can activate the estrogen receptor, tamoxifen inhibits parasite growth independently of this transcription factor. Tamoxifen is also a potent inducer of autophagy, and we find that the drug stimulates recruitment of the autophagy marker light chain 3-green fluorescent protein onto the membrane of the vacuolar compartment in which the parasite resides and replicates. In contrast to other antiparasitic drugs, including pimozide, tamoxifen treatment of infected cells leads to a time-dependent elimination of intracellular parasites. Taken together, these data suggest that tamoxifen restricts *Toxoplasma* growth by inducing xenophagy or autophagic destruction of this obligate intracellular parasite.

**IMPORTANCE** There is an urgent need to develop new therapies to treat microbial infections, and the repurposing of well-characterized compounds is emerging as one approach to achieving this goal. Using the protozoan parasite *Toxoplasma gondii*, we screened a library of 1,120 compounds and identified several compounds with significant antiparasitic activities. Among these were pimozide and tamoxifen, which are well-characterized drugs prescribed to treat patients with psychiatric disorders and breast cancer, respectively. The mechanisms by which these compounds target these disorders are known, but we show here that these drugs kill *Toxoplasma* through novel pathways, highlighting the potential utility of off-target effects in the treatment of infectious diseases.

## Abstract (precis)

There is an urgent need to develop new therapies to treat microbial infections, and the repurposing of well-characterized compounds is emerging as one approach to achieving this goal. Using the protozoan parasite *Toxoplasma gondii*, we screened a library of 1,120 compounds and identified several compounds with significant antiparasitic activities. Among these were pimozide and tamoxifen, which are well-characterized drugs prescribed to treat patients with psychiatric disorders and breast cancer, respectively. The mechanisms by which these compounds target these disorders are known, but we show here that these drugs kill *Toxoplasma* through novel pathways, highlighting the potential utility of off-target effects in the treatment of infectious diseases.

## INTRODUCTION

Infections with the obligate intracellular parasite *Toxoplasma gondii* lead to toxoplasmosis, which can cause significant morbidity and mortality primarily in individuals who were either infected *in utero* or postnatally infected and then became immunocompromised because of either disease or immunosuppressive therapies (1, 2). Infections of humans and other hosts occur by digestion of either tissue cysts (containing the bradyzoite form) in undercooked meat or oocysts (containing the sporozoite form) that are shed in felid fecal material. Once they are digested, the acidic environment of the stomach will release parasites that will go on to infect intestinal epithelial cells and transform into tachyzoites. Immune cells are recruited to the gut and are subsequently infected, and these infected cells are used by tachyzoites to disseminate to peripheral tissues (3). The resulting immune response or drugs used to treat toxoplasmosis can kill most of the disseminated parasites, although some can escape killing and form relatively quiescent tissue cysts (4). Tissue cyst reactivation in a healthy individual is usually asymptomatic because of this efficient immune response, but immunocompromised individuals are at risk of developing life-threatening disease.

Only a limited number of drugs are available to treat toxoplasmosis patients. The current treatment of choice is pyrimethamine and sulfadiazine, which acts by inhibiting parasite folate metabolism (5), and other treatments include atovaquone, which inhibits the cytochrome *bc* complex in the parasite mitochondrion (6), and clindamycin, which inhibits protein synthesis within the apicoplast (7), which is a relic plastid found in *Toxoplasma* and many other apicomplexan parasites. However, these drugs are poorly tolerated and cannot kill bradyzoites (8). In addition, resistance to these drugs can develop and vaccines are thus far ineffective in humans. Therefore, new treatments are needed.

Nonbiased screening of large libraries of compounds is a common approach to identifying lead compounds that can be further refined to develop novel therapeutics. While a lack of information regarding a compound’s host toxicity, mechanism of action, and pharmacokinetics are surmountable, addressing them is time consuming and costly. One approach to overcoming these challenges has been to test whether drugs currently prescribed to treat other conditions or compounds that are well-described inhibitors of specific pathways or processes have antiparasitic activity (3). In this work, we screened a library of ~1,100 known compounds to identify those that inhibit *Toxoplasma* growth. Among the compounds that we identified, we focused on pimozide and tamoxifen, which are well-characterized drugs that are currently prescribed to treat Tourette’s syndrome and breast cancer, respectively. We find that while both compounds effectively kill *Toxoplasma*, they do so via targets other than those thought to be their reported protein targets, indicating that they likely work through off-target mechanisms. Indeed, here we report that tamoxifen most likely kills *Toxoplasma* by inducing xenophagy, which is an autophagy-dependent mechanism for eliminating intracellular pathogens.

## RESULTS

### Small-molecule screen to identify known compounds that inhibit *T. gondii* growth.

The Tocriscreen Total library, which is a collection of 1,120 well-characterized small-molecule inhibitors, was screened to identify compounds that inhibited *T. gondii* growth. Thus, human foreskin fibroblasts (HFFs) plated in 96-well plates were pretreated with each compound at 5 µM and then infected with β-galactosidase (β-Gal)-expressing RH strain tachyzoites. After 72 h, the medium was removed and chlorophenol red–β-d-galactopyranoside (CPRG) was added to measure β-Gal activity. A standard curve generated within each plate was used to enumerate the parasites in each well. Although not designed to include known anti-*Toxoplasma* agents to serve as positive controls, the library did contain several established antiparasitic compounds such as artemisinin and mycophenolic acid that, as expected, significantly reduced parasite growth (9, 10). In addition, inhibitors of the transforming growth factor β (TGF-β) type I receptor family (LY364947 and SD208) also inhibited parasite growth, which is consistent with our earlier finding that another TGF-β type I receptor inhibitor, SB505124, potently reduced parasite growth (11). We did note that a third inhibitor of this receptor family, SB431542, did not affect parasite growth, most likely because it has a lower affinity for the receptor and thus its effective anti-*Toxoplasma* dose is higher than the dose used in the screen (12). These data therefore demonstrated the relative robustness of the assay.

**FIG 1:**
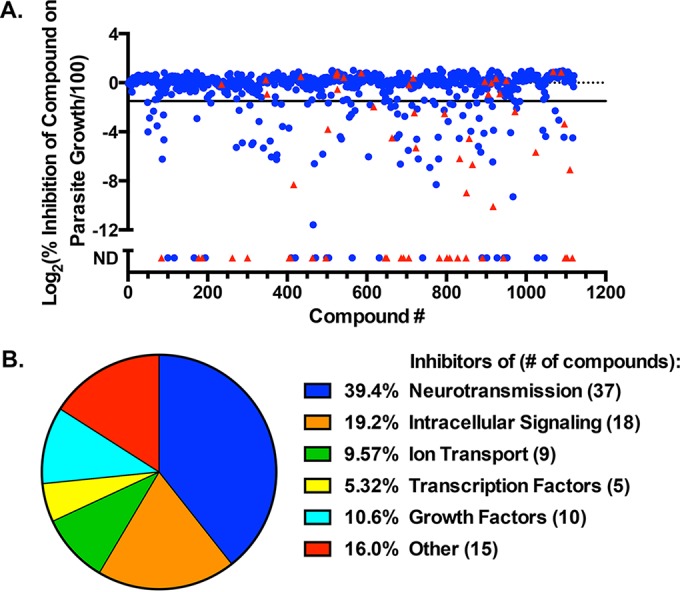
Small-molecule screen identifies compounds that inhibit *T. gondii* growth. (A) Graphic representation of screening data from Table S1 in the supplemental material. Blue squares, no host cell toxicity; red triangles, host cell toxicity. (B) Distribution of hits by cellular processes.

A total of 143 compounds reduced parasite growth by at least 3-fold (Fig. 1; see Table S1 in the supplemental material). Parallel host cell viability assays eliminated 37 of these compounds that negatively affected host cell viability from further consideration. Of the remaining 106 compounds, 98 were purchased separately to confirm their antiparasitic activity and to determine the 50% inhibitory concentration (IC_50_) of each compound for *Toxoplasma* tachyzoite growth. The secondary screen was performed at both 21% and 3% O_2_ because different host cell proteins and metabolic processes are important for *Toxoplasma* growth at tissue O_2_ levels (represented by 3% O_2_) compared to normoxic levels (21% O_2_) (13, 14), and optimal drugs would be those that killed at either O_2_ tension. A total of 94 compounds were confirmed to inhibit parasite growth at 21% O_2_, and only 11 of these had no effect on parasite growth at 3% O_2_. These drugs are known to target a large number of cellular processes, including neurotransmission, intracellular signaling, transcription, and ion channels (Fig. 1B; see Table S1 in the supplemental material).

10.1128/mSphere.00042-15.1 Table S1  The Primary Screening Data worksheet includes the drug screening data for the 1,120 compounds in Tocriscreen library genes that were screened. The Secondary Screening Data worksheet includes parasite and host cell growth data for the 98 compounds that were screened at 21% and 3% O_2_. Note that no IC_50_ indicates that the value was incalculable because there was no detectable decrease in growth. Download Table S1, XLSX file, 0.3 MB. Copyright © 2016 Dittmar et al. 2016 Dittmar et al. This content is distributed under the terms of the Creative Commons Attribution 4.0 International license.

### Dopaminergic inhibitors inhibit *Toxoplasma* growth.

**FIG 2:**
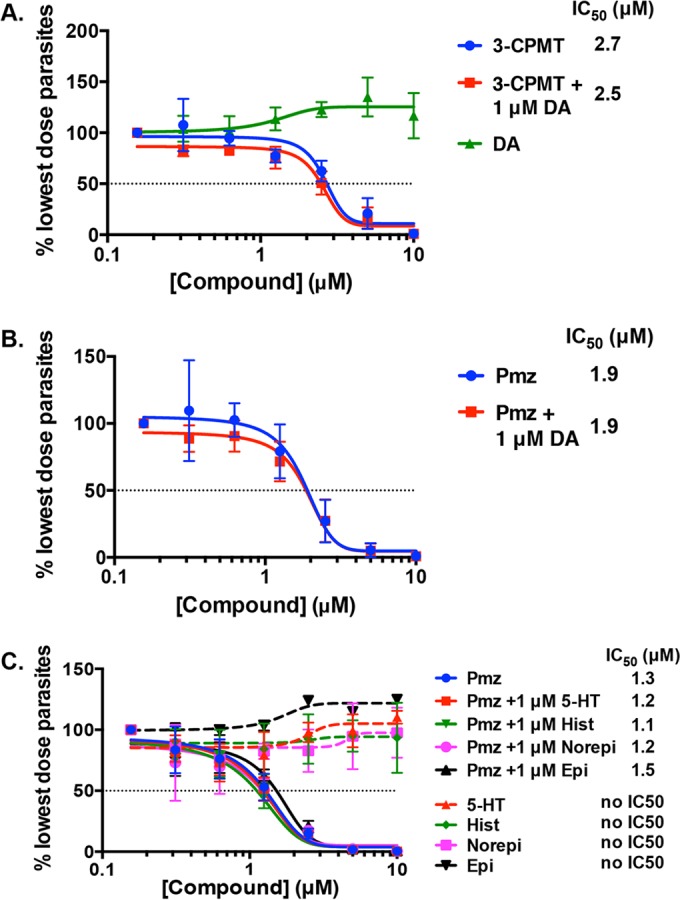
Dopaminergic inhibitors inhibit *Toxoplasma* growth. RH–β-Gal parasite growth was measured in HFFs treated with serial dilutions (0 to 10 µM) of 3-CPMT (A) or pimozide (Pmz) (B and C) in the absence or presence of the neurotransmitters indicated (1 µM). DA, dopamine; 5-HT, 5-hydroxytryptamine; Hist, histamine; Norepi, norepinephrine; Epi, epinephrine.

A significant number of compounds that target dopaminergic signaling were identified in our screen, which was intriguing since *Toxoplasma* has been proposed to alter host dopaminergic signaling for growth (15, 16). Therefore, we examined two different classes of these inhibitors, 3-CPMT, a dopamine reuptake inhibitor (IC_50_ = 2.1 µM), and pimozide (IC_50_ = 1.8 µM), a dopamine D2 receptor antagonist. *Toxoplasma* has been proposed to use dopamine for growth (17), and we hypothesized that 3-CPMT affects parasite growth by reducing host cell dopamine levels by blocking its reuptake from the extracellular milieu. Thus, we tested whether addition of 1 µM dopamine (>30× the *K_i_* of 3-CPMT for the dopamine transporter [18]) enhanced parasite growth and found that the neurotransmitter neither increased the IC_50_ of 3-CPMT nor significantly increased parasite growth on its own (Fig. 2A). We next tested whether exogenous dopamine could reverse pimozide inhibition of parasite growth due to the drug’s inhibition of dopamine receptor signaling. Similar to its effect on 3-CPMT, 1 µM dopamine (~500 times its IC_50_ for dopamine receptor signaling [19]) did not affect the ability of pimozide to impact parasite replication (Fig. 2B). Pimozide is also reported to inhibit serotonergic, histaminergic, and noradrenergic signaling (20, 21). Addition of serotonin, histamine, norepinephrine, or epinephrine did not affect the sensitivity of *Toxoplasma* to pimozide (Fig. 2C). Together, these data indicate that off-target effects of 3-CPMT and pimozide are the basis of their inhibition of *Toxoplasma*. Since pimozide has a lower IC_50_ than 3-CPMT, the remaining experiments were performed only with pimozide.

### Pimozide inhibits *Toxoplasma* invasion and replication.

**FIG 3:**
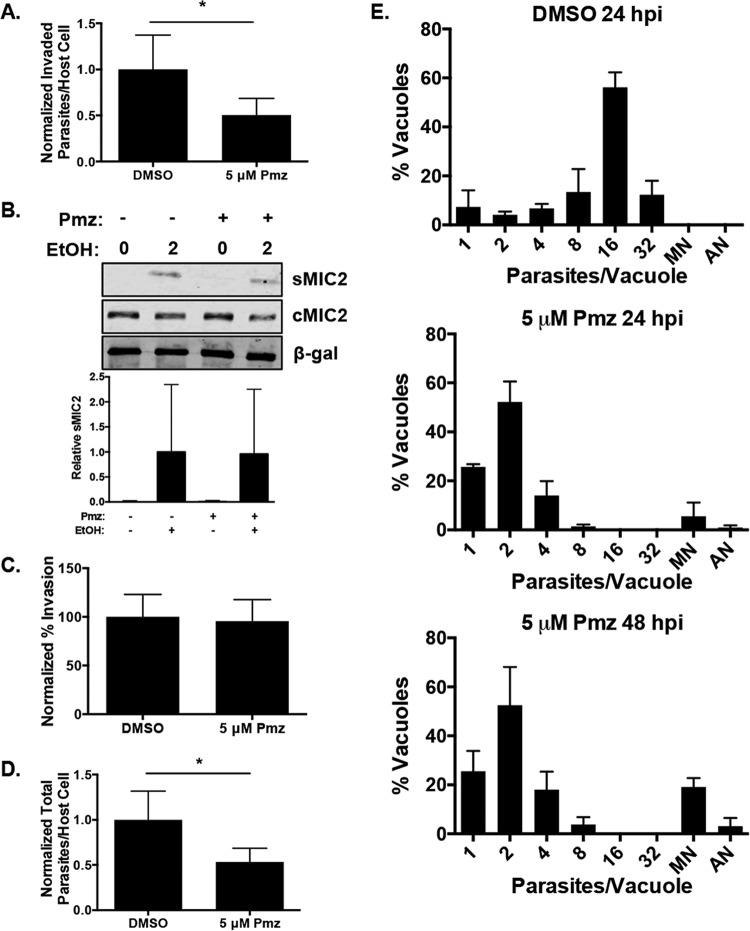
Pimozide inhibits parasite invasion and replication. (A) Host cell monolayers were pretreated with 5 µM pimozide (Pmz) or DMSO for 1 h prior to infection with RH–β-Gal–GFP parasites for 1 h. The cells were fixed without permeabilization and stained with DAPI (to identify host cell nuclei) and anti-SAG1 antiserum. Invasion events were scored via differential staining as GFP^+^ SAG1^−^ (invaded) and GFP^+^ SAG1+ (extracellular). A minimum of 500 host cells were counted for each duplicate sample. Shown are the average values and standard deviations from one experiment representative of three independent experiments performed in duplicate. (B) DMSO- or pimozide-treated extracellular RH–β-Gal–GFP tachyzoites were incubated in the absence or presence of 1% ethanol (EtOH) for 2 min at 37°C. The supernatants (sMIC2) and parasites (cMIC2) were Western blotted to detect MIC2. β-Gal was detected as a loading control. Quantification of sMIC from three independent experiments was performed. (C) The percentage of intracellular parasites from panel B was calculated to determine invasion efficiency. (D) The total numbers of parasites per host cell in DMSO- and pimozide-treated cells were compared. (E) RH parasites were allowed to invade HFFs, and 2 h later, DMSO or pimozide was added. After 24 and 48 h, the cells were fixed and stained with anti-SAG1 antiserum and the number of parasites per vacuole was determined. *, *P* < 0.05 (unpaired Student *t* test). MN, multinucleated; AN, anucleated.

*Toxoplasma* replicates via a lytic cycle composed of repeated rounds of invasion, replication, and egress (22). *Toxoplasma* invasion is a highly coordinated process in which invasion is started by parasites attaching to the host cell through a low-affinity interaction between unidentified parasite and host factors. An unknown trigger then induces the calcium-dependent release of micronemal proteins that act as adhesins that form an intimate attachment between the parasite and the host cell. Finally, the parasite traverses the surface of the host cell until it begins to penetrate the host cell while simultaneously forming the nascent parasitophorous vacuole (PV) (23). To test whether pimozide affected parasite invasion, host cells were pretreated with pimozide or the vehicle control for 60 min and then RH–β-Gal–green fluorescent protein (GFP) parasites were added in the presence of pimozide or the vehicle control, respectively. After 60 min, the cells were fixed but not permeabilized and then stained with anti-SAG1 antiserum to discriminate between intracellular (GFP^+^ SAG1^−^) and extracellular (GFP^+^ SAG1^+^) parasites. We found that pimozide significantly reduced the number of intracellular parasites by ~50% (Fig. 3A). Pimozide had no apparent effect on the ability of ethanol to induce calcium-dependent secretion of the MIC2 micronemal protein (24) or on the ratio of intracellular to extracellular parasites (Fig. 3B and C), indicating that the drug did not affect the steps involved in intimate attachment or host cell penetration. In contrast, pimozide reduced the total number of parasites associated with the host cell by ~50% (Fig. 3D), indicating that the drug affected the initial step in parasite invasion, which is the loose association of the parasite with the host plasma membrane.

We next examined the effect of pimozide on parasite replication by allowing parasites to invade host cells for 120 min. The cells were then washed to remove extracellular parasites, and then fresh medium containing either the vehicle or pimozide was added. The cells were fixed 24 h later and then stained to detect SAG1 and host nuclei with 4′,6-diamidino-2-phenylindole (DAPI). In contrast to vehicle-treated parasites that largely consisted of vacuoles containing 8 and 16 parasites, the pimozide-treated parasites contained only 2 parasites per vacuole (Fig. 3E). When the infection was allowed to continue for 48 h (a time point at which vehicle-treated parasites could not be counted because they had already lysed out), the pimozide-treated parasites did not progress from 2 parasites per vacuole, indicating that the drug arrested parasite replication rather than slowed it. We also found that ~5% and 19% of the vacuoles contained multinucleated parasites following treatment for 24 and 48 h, respectively, indicating a slight but statistically significant (*P* < 0.05) time-dependent effect on cytokinesis.

### Tamoxifen inhibits *Toxoplasma* invasion and replication.

The Tocriscreen library contains six compounds that are established inhibitors of estrogen receptor (ER) signaling, and four of these (tamoxifen [IC_50_ = 1.9 µM], fERB033 [IC_50_ = 3.1 µM], raloxifene [IC_50_ =2.0 µM], and Y134 [IC_50_ = 3.5 µM]) were identified in our screen as *Toxoplasma* growth inhibitors. Identification of these estrogen antagonists was intriguing because tamoxifen and raloxifene are well-studied drugs currently used to treat breast cancer and osteoporosis patients, respectively. In addition, estrogen was previously shown to increase numbers of *Toxoplasma* tissue cysts in the brains of parasite-infected mice (25). Since tamoxifen is the best-characterized antiestrogen inhibitor identified in this screen, we performed the next experiments with tamoxifen.

**FIG 4:**
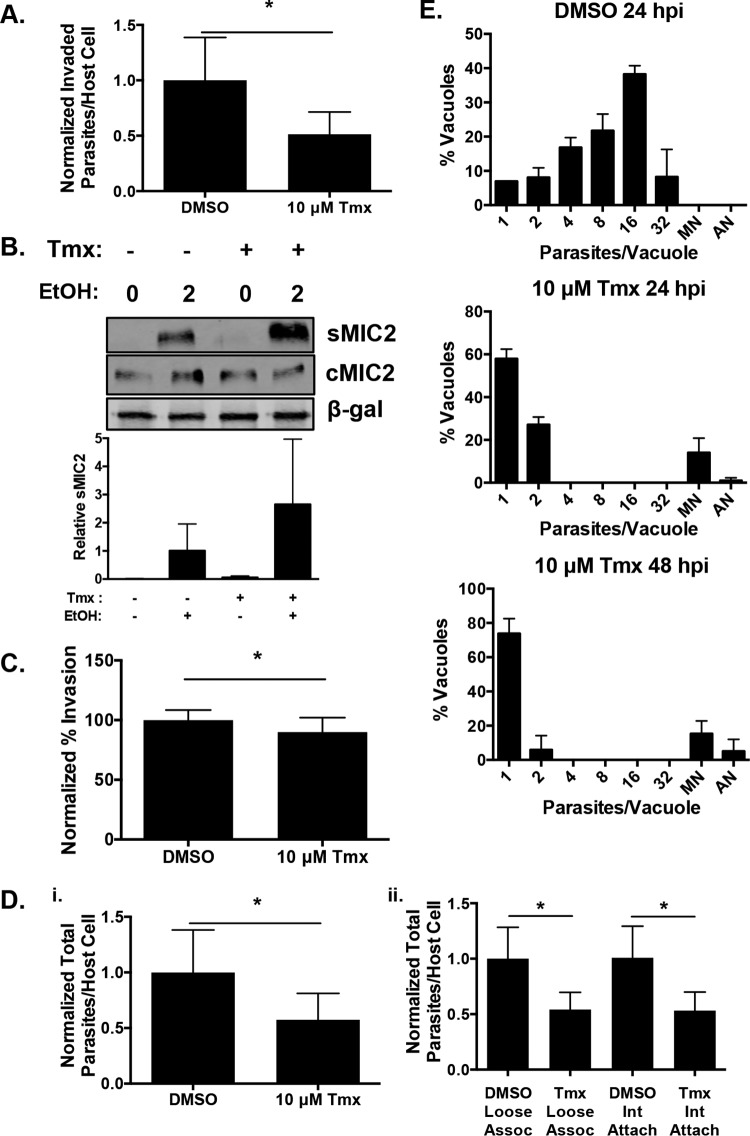
Tamoxifen inhibits parasite invasion and replication. (A) Host cell monolayers were pretreated with 10 µM tamoxifen (Tmx) or DMSO for 1 h prior to infection with RH–β-Gal–GFP parasites for 1 h. The cells were fixed without permeabilization and stained with DAPI and anti-SAG1 antiserum. Invasion was assessed as GFP^+^ SAG1^−^ (invaded) and GFP^+^ SAG1+ (extracellular) parasites. A minimum of 500 host cells were counted for each duplicate sample. Shown are average values and standard deviations from an experiment representative of three independent experiments. (B) DMSO- or tamoxifen-treated extracellular RH–β-Gal–GFP tachyzoites were incubated in the absence or presence of 1% ethanol (EtOH) for 2 min at 37°C. The supernatants (sMIC2) and parasites (cMIC2) were Western blotted to detect MIC2. β-Gal was detected as a loading control. Quantification of sMIC from three independent experiments was performed. (C) The percentages of intracellular and extracellular parasites from panel A were calculated to determine invasion efficiency. (Di) The total numbers of parasites per host cell in DMSO- and tamoxifen-treated cells were compared. (Dii) Shown are the differences in the total number of parasites per host cell that are loosely associated or intimately (Int) attached to the host cell in DMSO- and tamoxifen-treated samples. (E) RH parasites were allowed to invade HFFs, and DMSO or tamoxifen was added 2 h later. After 24 and 48 h, the cells were fixed and stained with anti-SAG1 antiserum and the number of parasites per vacuole was determined. *, *P* < 0.05 (unpaired Student *t* test). MN, multinucleate; AN, anucleate.

First, we sought to determine which step of the parasite’s lytic growth cycle is affected by tamoxifen. To test whether tamoxifen reduces parasite invasion, host cells were mock treated or pretreated for 60 min with 10 µM tamoxifen and then freshly egressed parasites were added to the host cells and invasion was allowed to proceed for 60 min in the continued absence or presence of tamoxifen. The cells were then fixed and stained to discriminate between intracellular and extracellular parasites, and numbers of intracellular parasites per host cell were determined. We found that tamoxifen significantly reduced the numbers of parasites that invaded cells by approximately 55% (Fig. 4A).

Next, we sought to determine the specific step in invasion that was affected by tamoxifen. First, we assessed the effect of tamoxifen on microneme secretion by examining ethanol-induced release of MIC2 from purified extracellular parasites (24). We found that the drug increased ethanol-induced MIC2 secretion by ~2-fold (Fig. 4B). We next assessed invasion efficiency by calculating the percentage of parasites per field that had penetrated the host cell in the absence or presence of tamoxifen and found a slight but statistically significant difference (Fig. 4C). Finally, we determined the total (intracellular and extracellular) number of parasites associated with host cells to determine if the drug interfered with the initial interaction between *Toxoplasma* and the host cell. The data indicated that tamoxifen reduced this ratio by ~50% (Fig. 4Di). We further studied this initial interaction by assessing both loosely associated parasites and intimately attached parasites after treatment with dimethyl sulfoxide (DMSO) or tamoxifen and found the same change in the total number of parasites associated with host cells under both conditions (Fig. 4Dii). Taken together, these data indicate that, similar to pimozide, tamoxifen reduces parasite invasion primarily by limiting the initial contact between *Toxoplasma* and host cells. Because tamoxifen is a reversible inhibitor (26 – 28), we could not determine whether the drug inhibits *Toxoplasma* invasion by inhibiting either a host or a parasite target.

We next tested the effect of tamoxifen on parasite replication by allowing parasites to invade HFFs in the absence of tamoxifen and then adding the drug or vehicle control 120 min later and allowing the parasites to continue to grow for an additional 24 or 48 h. At each time point, the cells were fixed and numbers of parasites per vacuole were determined. In contrast to vehicle-treated parasites, tamoxifen significantly reduced parasite growth at 24 hpi with ~58% and 24% of the vacuoles from the tamoxifen-treated samples containing only one or two parasites, respectively. The majority of the remaining vacuoles contained single multinucleated SAG1^+^ parasites (Fig. 4D). At 48 hpi, the proportion of vacuoles containing a single parasite increased to 75% with a concomitant decrease in vacuoles with two parasites. Together, these data indicate that tamoxifen blocks *Toxoplasma* growth primarily by limiting parasite replication and that its effects on invasion are secondary.

### Tamoxifen inhibits parasite growth independently of estrogen signaling.

**FIG 5:**
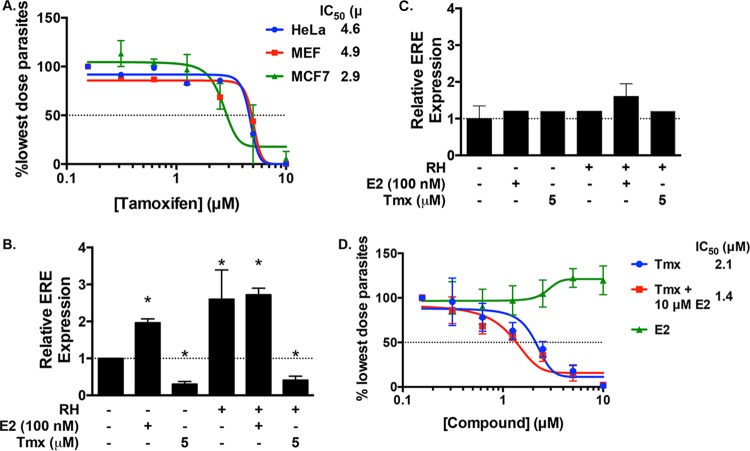
Tamoxifen inhibits *Toxoplasma* growth independently of estrogen signaling. (A) ER-negative (HeLa and MEF) and ER-positive (MCF7) cells in 96-well plates were infected with RH–β-Gal–GFP parasites and treated with increasing doses of tamoxifen (0 to 10 µM). After 72 h, parasite growth was assessed by measuring β-Gal activity. (B, C) MCF7 (B) and MEF (C) cells were transfected with an ER element (ERE) luciferase reporter and then treated as indicated. After 18 h, lysates were collected and luciferase activity was measured. (D) HFFs in 96-well plates were infected with RH–β-Gal–GFP parasites and treated with increasing doses of tamoxifen (in the absence or presence of 10 µM estrogen [E2]) or estrogen. Parasite growth was measured after 72 h by the CPRG assay. *, *P* < 0.05 (unpaired Student *t* test).

Estrogen binding to the ER induces a conformational change in the receptor that allows the ER to bind DNA and other transcriptional regulatory proteins (29). There are two ER isoforms whose expression varies among cell types (30). Expression of these ER isoforms is either undetectable or nominal in the HFFs used for the screen, suggesting that the drug functions in an ER-independent manner (31, 32). We tested this by assessing the growth of β-Gal-expressing parasites in tamoxifen-treated MCF7 cells, which express the ER (32), and in HeLa cells and murine embryonic fibroblasts (MEFs) that, like HFFs, are ER deficient or express the ER at significantly lower levels than MCF7 cells do (33, 34). We found that parasite growth in all three cell types was similarly affected by tamoxifen, although the IC_50_ in MCF7 cells was slightly lower (Fig. 5A).

One way that *Toxoplasma* alters host cell signaling and gene expression is by injecting parasite-encoded effector proteins into the cytosol of the host cell (35). However, the *Toxoplasma* genome database (http://www.toxodb.org) does not predict the presence of an apparent *Toxoplasma*-encoded ER homologue. Regardless, we tested whether tamoxifen was inhibiting a factor secreted into the host that was either acting to upregulate a host ER isoform or was functioning as a parasite-encoded ER homologue. Thus, MCF7 cells and MEFs were transfected with a plasmid in which the luciferase reporter is cloned downstream of an ER-responsive element. The cells were then mock treated, treated with estrogen (as a positive control), or infected with *Toxoplasma* tachyzoites at a multiplicity of infection (MOI) of 4 and grown in the absence or presence of tamoxifen. After 18 h, the cells were lysed and luciferase activity was measured. As expected, estrogen led to a 2-fold increase in ER activity in MCF7 cells (Fig. 5B). *Toxoplasma* similarly increased ER activity in MCF7 cells, indicating that the parasite can activate the ER and this increase was tamoxifen sensitive, but neither estrogen nor *Toxoplasma* increased luciferase reporter activity in the ER-deficient MEFs (Fig. 5C).

Tamoxifen can also inhibit the ability of estrogen to bind and activate other proteins such as the G protein-coupled receptor GPR30 (36). To assess whether tamoxifen reduced parasite growth by inhibiting a distinct estrogen binding protein, we tested whether exogenous estrogen could decrease the sensitivity of *Toxoplasma* to tamoxifen in ER-deficient HFFs. Thus, RH–β-Gal growth was measured in cells treated with increasing concentrations of tamoxifen in the absence or presence of 10 µM estrogen, which is ~300 times the IC_50_ of tamoxifen (37). The data indicated that estrogen did not alter the sensitivity of *Toxoplasma* to tamoxifen, nor did it significantly enhance parasite growth on its own (Fig. 5D).

### Tamoxifen induces xenophagy in *Toxoplasma*-infected cells.

**FIG 6:**
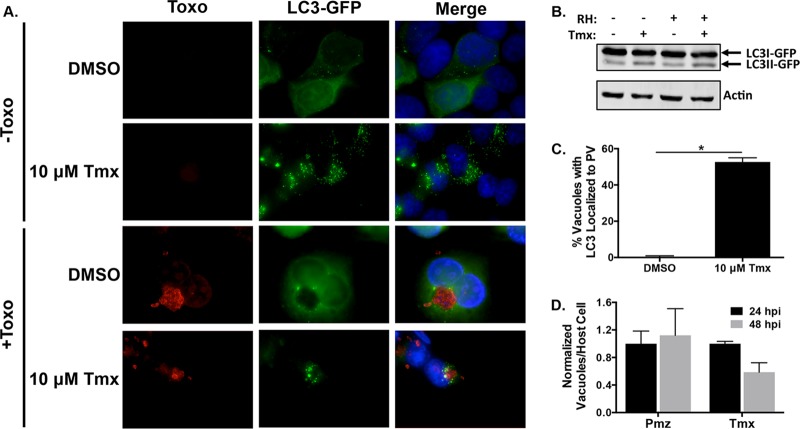
Tamoxifen induces accumulation of LC3-GFP on the PV. (A) MCF7 cells were transfected with an LC3-GFP expression construct and then either mock or parasite infected. After 2 h, tamoxifen (Tmx; 10 µM) or DMSO was added and the cells were grown for an additional 24 h and then fixed and stained to detect SAG1. Toxo, *Toxoplasma*. (B) Western blot analysis of LC3-GFP expression and the amount of the LC3II isoform of LC3-GFP and the extent of autophagy induction (LC3II). Shown is a blot representative of three independent experiments. (C) Quantification of LC3-GFP accumulation on the PV in parasite-infected cells treated with either DMSO or tamoxifen. (D) Cells were infected with *Toxoplasma* and 2 h later treated with either 10 µM tamoxifen or 5 µM pimozide as a control for 24 and 48 h. The number of vacuoles per host cell was determined at each time point. A minimum of 300 host cells per experiment were counted. Shown are the average values and standard deviations of three (tamoxifen) and two (pimozide) independent experiments, respectively. *, *P* < 0.05 (paired Student *t* test).

**FIG 7:**
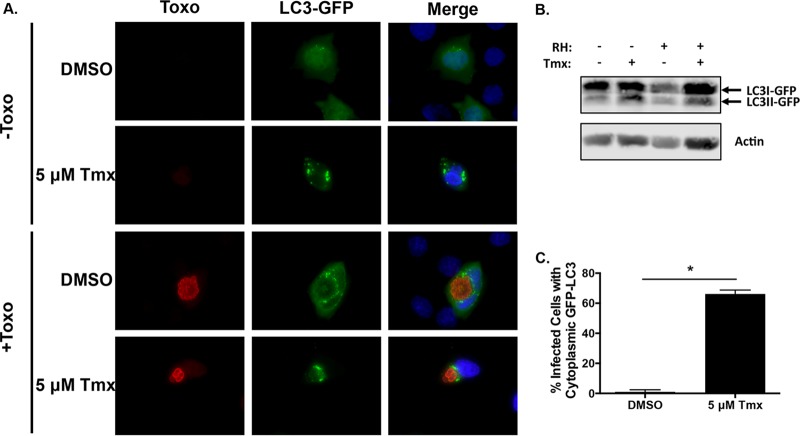
Tamoxifen induces accumulation of LC3-GFP on the PVM in murine cells. (A) MEFs were transfected with the LC3-GFP expression construct and then either mock or parasite infected. After 2 h, tamoxifen (Tmx; 5 µM) or DMSO was added and the cells were grown for an additional 24 h and then fixed and stained to detect SAG1. Toxo, *Toxoplasma*. (B) Western blot analysis shows levels of LC3-GFP expression and the extent of autophagy induction. Shown is a blot representative of three independent experiments. (C) Quantification of LC3-GFP accumulation on the PVM in parasite-infected cells that were treated with either DMSO or tamoxifen. *, *P* < 0.05 (paired Student *t* test).

Besides activating the ER and GPR30, tamoxifen also induces autophagy by increasing cellular ceramide levels (38). Likewise, a second ER antagonist, raloxifene, which is structurally related to Y134 (both of these compounds were identified in our screen), induces autophagy (39). The Tocris library contained two other ER antagonists that did not reduce *Toxoplasma* growth—ICI182780, whose induction of autophagy has been debated but appears to be ER dependent (40, 41), and ZK164015, which is not structurally related to tamoxifen or raloxifene. The fourth ER inhibitor identified in our screen, fERB033, has not, to our knowledge, been examined for an ability to activate autophagy. Because autophagy induced by gamma interferon (IFN-γ) and CD40 is a key cellular defense against *Toxoplasma* (42 – 45), we hypothesized that tamoxifen restricts parasite growth by inducing autophagic degradation of *Toxoplasma*. This hypothesis was first explored by testing whether tamoxifen induced the accumulation of microtubule-associated protein 1 light chain 3 (LC3) with the PV, which represents a late and committed step in autophagosome-mediated destruction of the PV (43, 46). Our initial studies with an anti-LC3 antibody indicated cross-reactivity with intracellular parasites (not shown). Therefore, we were concerned that, following tamoxifen treatment of *Toxoplasma*-infected host cells, *Toxoplasma* LC3 would be released from the dying parasites and be detected by immunocytochemistry in the host cell cytoplasm. Because HFFs are transfected with low efficiencies, ER-positive MCF7 cells were transiently transfected with an LC3-GFP expression construct and then mock or parasite infected for 2 h, at which time tamoxifen or DMSO was added. The parasites were grown for 24 h, fixed, stained with anti-SAG1 antiserum, and visualized to assess LC3-GFP recruitment to the PV membrane (PVM). As expected, tamoxifen induced the accumulation of LC3-GFP puncta in mock-infected cells and also increased the amount of the lipidated form of LC3 (LC3II) (Fig. 6A and B). Tamoxifen triggered LC3-GFP accumulation on the PVM (defined as ≥50% of the PVM-associated LC3-GFP) (Fig. 6C) and also increased LC3II abundance in parasite-infected cells. This change in LC3-GFP localization is most likely not due to a change in LC3-GFP expression following tamoxifen treatment since its overall abundance did not significantly increase in the drug-treated cells (Fig. 6B). A similar effect of tamoxifen on LC3-GFP expression and localization was observed in *Toxoplasma*-infected MEFs (Fig. 7).

The tamoxifen-induced association of LC3-GFP with the PV suggested that the drug was restricting parasite growth at least in part by inducing autophagic degradation of the PV. We were precluded from directly testing this model with cells lacking components of the autophagy machinery since autophagy is a cellular response required for cells to survive tamoxifen treatment and because pharmacological inhibitors of autophagy, such as 3-methyladenine, restrict parasite growth (47, 48). We therefore hypothesized that if tamoxifen was inducing autophagic degradation of intracellular parasites, then a time-dependent decrease in the numbers of vacuoles per host cell would be observed. For these experiments, we could not use MEFs or MCF7 cells because, as transformed cells, they would continue to replicate during the course of the experiments. Therefore, we infected confluent HFFs, whose growth is contact inhibited, with *Toxoplasma* and added tamoxifen 2 h later. The cells were grown for an additional 24 and 48 h, at which time the cells were fixed and stained with anti-SAG1 antiserum and DAPI and the numbers of vacuoles per host cell were determined. The data indicated that compared to 24 hpi, there was an almost 50% reduction in the numbers of vacuoles per host cell at 48 hpi (Fig. 6D). This decrease in the numbers of vacuoles was likely not a result of the parasite prematurely egressing from the host cell because numbers of HFFs did not appear to decrease and extracellular parasites were not observed. Together, these data suggest that tamoxifen controls parasite growth by inducing autophagic degradation of intracellular parasites, which is a process known as xenophagy.

## DISCUSSION

Our screen identified 94 new compounds with potent antiparasitic activities. Some compounds in the Tocriscreen library are established anti-*Toxoplasma* agents (such as mycophenolic acid, compound 1, and artemisinin) and these reduced parasite growth (9, 10, 49). On the other hand, 3-hydroxy-3-methyl-glutaryl coenzyme A reductase inhibitors, such as mevastatin and lovastatin, did not reduce parasite growth, which is consistent with the finding that statins kill *Toxoplasma* only when the parasite cannot synthesize its own isoprenoids (50). Together, these compounds effectively served as controls to establish the specificity of our screen.

One advantage of drug repurposing screens is that pharmacokinetic and pharmacodynamic parameters are established for each compound and their putative target(s) is also known (51, 52). Together, these properties are expected to accelerate their novel clinical uses; thalidomide’s success in treating erythema nodosum leprosum and multiple myeloma patients is just one example of the potential that drug repurposing has in the treatment of human diseases (52). Tamoxifen and pimozide were specifically selected for follow-up experiments because they are well-characterized drugs used to treat breast cancer and neurological disorder patients, respectively. Even though molecular targets of each drug are known, our studies demonstrated that both compounds blocked parasite growth through novel targets. Identification of these targets is critical in order to consider the off-label use of tamoxifen, pimozide, and other drugs that we screened, and therefore, we believe that caution must be taken before data from this screen and others can be directly applied to clinics.

Our interest in pimozide as an inhibitor of dopamine signaling was spawned by earlier studies suggesting that host dopamine signaling was modulated by infection although direct biochemical data demonstrating that dopamine receptor activation following infection was lacking (53, 54). It has also been proposed that *Toxoplasma* synthesizes dopamine on its own and that this promotes parasite growth (17, 55), although this has been controversial (56, 57). We predicted that if pimozide blocks parasite growth by inhibiting dopamine receptors, then exogenously applied dopamine would compete with pimozide and alter the drug’s IC_50_. This was not the case, and in contrast to other work (17), we also found that dopamine had only a small effect on parasite growth at high concentrations. Thus, pimozide most likely impacts parasite growth independently of its function as an antagonist of dopamine and other neurotransmitters, although whether the drug acts on a host- or parasite-encoded target is not known. One approach to identifying a target for pimozide would be the isolation of drug-resistant mutants and identification of pimozide resistance genes by whole-genome sequencing. We used this approach in the past to identify a parasite mitogen-activated protein kinase as a target for SB505124, which is a kinase inhibitor (58). In contrast, our repeated attempts to isolate pimozide-resistant mutants were unsuccessful (data not shown), suggesting that the drug’s effect on *Toxoplasma* is complex.

Our collective data indicate that tamoxifen inhibits *Toxoplasma* replication via a mechanism independent of its ability to antagonize ER signaling even though we found that *Toxoplasma* activates ER-dependent transcription. In addition, we showed that tamoxifen reduced the overall number of parasite vacuoles and also induced the accumulation of LC3-GFP on the PVM. Together, these data point to a mechanism by which tamoxifen kills *Toxoplasma* by inducing xenophagy. Xenophagy is now a well-recognized mechanism used by IFN-γ and CD40 to control *Toxoplasma* replication (43, 44, 59), and our work represents the first example of non-immune-stimulated xenophagy to control *Toxoplasma* growth and appears not to be restricted by the host species, but more in-depth biochemical and ultrastructural analyses of both the parasite and the host cell are needed to better define the mechanisms regulating xenophagy in parasite-infected host cells. In addition, it will be important to determine whether there are host species-specific pathways involved since human and murine cells differentially execute IFN-γ-dependent autophagy (60). These assays will require cells that do not activate autophagy upon exposure to tamoxifen. However, loss of autophagy genes leads to cell death after the addition of tamoxifen, indicating that autophagy is a stress response required to prevent tamoxifen-induced death (47). We also cannot rule out the possibility that tamoxifen arrests parasite replication independently of xenophagic vacuole elimination. Similarly, it is unclear whether the effect of tamoxifen on parasite invasion is related to its induction of autophagy, and future work will address these issues.

In summary, we identified 94 compounds that potently inhibit parasite growth. Although several of these inhibitors have been established to modulate estrogen and dopamine signaling pathways, they inhibited parasites independently of these pathways. We conclude that while drug repurposing screens are useful and have the potential to quickly impact patient care, target confirmation is needed since off-target effects can be significant and misleading.

## MATERIALS AND METHODS

### Cells and parasites.

All *Toxoplasma* strains were maintained by serial passage on confluent monolayers of HFFs in Dulbecco’s modified Eagle’s medium (DMEM) supplemented with 10% fetal bovine serum, glutamine, and penicillin-streptomycin. For all experiments, parasites were released from nonlysed host cells by passage through a 27-gauge syringe needle three times, followed by extensive washing in DMEM. The other host cells were propagated in the same culture medium. All host cells and parasites were routinely tested and found to be negative for *Mycoplasma* contamination as previously described (58).

### Small-molecule screen.

The Tocriscreen compound library (Tocris, Bristol, United Kingdom), consisting of 1,120 compounds (each stored as a 10 mM stock solution in DMSO), was added to confluent monolayers of HFFs in 96-well plates at a final concentration of 5 µM by automated pin transfer. Within 2 h after the addition of the drug, HFFs were infected with RH strain β-Gal–GFP-expressing (RH–β-Gal–GFP) parasites (from Gustavo Arrizabalaga, Indiana University) at an MOI of 1:5 (parasite/host cell ratio) and allowed to grow for 72 h before measurement of parasite growth with the β-Gal substrate CPRG (13). The number of parasites in each well was determined by linear extrapolation of standard curves generated in parallel in each plate. The effect of each compound on the viability of uninfected host cells was determined with the Cell Titer Blue viability assay (Promega, Madison, WI) after 72 h of growth. Average values and standard deviations of host cell growth were calculated, and those compounds that reduced host cell viability by 1.5 standard deviations were removed from further analysis. Follow-up assays with HFFs, MEFs, and MCF7 cells were performed under the same infection conditions.

### Parasite invasion and replication assays.

*Toxoplasma* invasion assays were performed essentially as previously described (61). Briefly, RH–β-Gal–GFP parasites were added to HFFs (MOI of 3:1) plated on coverslips that were pretreated with either the drug or the vehicle for 1 h. Sixty minutes after the parasites were added, the medium was gently removed and the cells were fixed with 3% paraformaldehyde. Parasites were stained with rabbit anti-SAG1 antiserum (from John Boothroyd, Stanford University) and detected with anti-rabbit Alexa Fluor 594 (Invitrogen, Carlsbad, CA) without permeabilization of the cells. Coverslips were mounted in Vectashield containing DAPI (Vector Labs, Burlingame, CA). Intracellular parasites were scored as GFP^+^ SAG1^−^, and extracellular parasites were scored as GFP^+^ SAG1^+^. A total of 20 randomly selected fields were counted per condition.

Parasite replication was measured by adding parasites (MOI of 1:4) to confluent HFFs on coverslips and allowing them to invade for 2 h, at which time drugs or vehicle controls were added. At the times indicated, cells were fixed and stained with anti-SAG1 antiserum and the number of parasites per vacuole was determined. A minimum of 50 vacuoles per coverslip were counted.

### Microneme secretion assay.

Purified RH–β-Gal–GFP parasites were resuspended in Hanks balanced salt solution supplemented with 10 mM HEPES (pH 7.4). After the parasites were pretreated for 10 min with either the drug or the vehicle, secretion was induced by the addition of ethanol to 1% for 2 min at 37°C and then the cells were placed on ice for 5 min (24). Supernatants were collected by centrifugation at 2,000 × *g*, SDS-PAGE sample buffer was added to the supernatants and pellets, and then they were boiled for 3 min. The samples were Western blotted to detect MIC2 (anti-MIC2 antibody was provided by Vern Carruthers, University of Michigan) and β-Gal (Promega). All blots were imaged with the Odyssey Clx Imaging System (LI-COR, Lincoln, NE) and quantified by the LI-COR software.

### Luciferase assay.

MEFs or MCF7 cells were transiently transfected with 3× ERE TATA luc (Addgene plasmid no. 11354) and phRL-TK (Promega) for 24 h. Cells were then either mock infected or infected with *Toxoplasma* (MOI of 4) and treated with the vehicle or estrogen (100 nM) and/or tamoxifen (5 µM). After 24 h, ER element activity was assessed with the Dual-Glo luciferase assay system (Promega).

### LC3-GFP assays.

MCF7 cells and MEFs were transiently transfected with an LC3-GFP expression plasmid (62) (kindly provided by William Jackson, University of Maryland) as previously described (13). The cells were infected with RH strain parasites after 24 h, and either tamoxifen or DMSO was added 2 h later. The cells were grown for an additional 24 h, at which time they were fixed and stained with rabbit anti-SAG1 antiserum and DAPI. All images were processed similarly, at least 50 images per condition were analyzed, and the experiments were repeated at least two independent times. LC3-GFP accumulation on the PVM was defined as staining associated with >50% of the PVM. Western blot assays of lysates prepared from the LC3-GFP-transfected cells were performed with anti-GFP and anti-actin antibodies (Cell Signaling).
